# The role of the human gut microbiota in colonization and infection with multidrug-resistant bacteria

**DOI:** 10.1080/19490976.2021.1911279

**Published:** 2021-04-18

**Authors:** Irene Wuethrich, Benedikt W. Pelzer, Yascha Khodamoradi, Maria J. G. T. Vehreschild

**Affiliations:** a Department of Biosystems Science and Engineering, ETH Zurich, Basel, Switzerland; b Center for Integrated Oncology Aachen Bonn Cologne Duesseldorf, Department I of Internal Medicine, University of Cologne, Cologne, Germany; c Department of Internal Medicine, Infectious Diseases, University Hospital Frankfurt, Goethe University Frankfurt, Frankfurt Am Main, Germany

**Keywords:** Microbiota, multidrug resistance, bacterial infection, dysbiosis, microbial therapy

## Abstract

About 100 years ago, the first antibiotic drug was introduced into health care. Since then, antibiotics have made an outstanding impact on human medicine. However, our society increasingly suffers from collateral damage exerted by these highly effective drugs. The rise of resistant pathogen strains, combined with a reduction of microbiota diversity upon antibiotic treatment, has become a significant obstacle in the fight against invasive infections worldwide.

Alternative and complementary strategies to classical “Fleming antibiotics” comprise microbiota-based treatments such as fecal microbiota transfer and administration of probiotics, live-biotherapeutics, prebiotics, and postbiotics. Other promising interventions, whose efficacy may also be influenced by the human microbiota, are phages and vaccines. They will facilitate antimicrobial stewardship, to date the only globally applied antibiotic resistance mitigation strategy.

In this review, we present the available evidence on these nontraditional interventions, highlight their interaction with the human microbiota, and discuss their clinical applicability.

## Introduction

The human body harbors a multitude of microorganisms, including bacteria, fungi, archaea, and viruses, which exist in a symbiotic relationship with their host. The entirety of these commensals is referred to as the microbiota, and their collective genomic information as the human microbiome.^1,2^ Within the microbiota, bacteria play a central role. All body surfaces are characterized by their specific bacteriome, which describes the bacterial component of the microbiome. Over 2000 bacterial species have been identified as human commensals, a majority of which remain uncultured.^3^ The gut microbiota composition varies between individual persons and has been found to consist of a few hundred bacterial operational taxonomic units (OTU) on average.^4–6^ They constitute a subset of the overall phylogenetic diversity found in the corresponding human population.^7–9^ Most of these bacteria reside in the colon^10^ and occupy different functional niches.^3^

**Figure 1.**
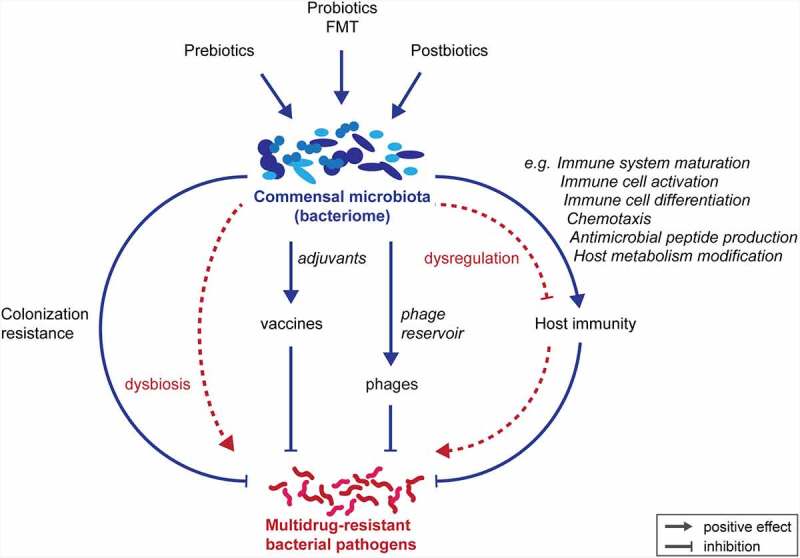
Intervention strategies against multidrug-resistant bacterial pathogens that are mediated or boosted by healthy commensal microbiota. In cases of dysbiosis or dysregulation, the microbiota may also contribute to increased pathogen colonization and disease. FMT: fecal microbiota transfer

The development of high-throughput sequencing techniques has improved our understanding of the role our commensal bacteria play in maintaining human homeostasis. Their regulatory properties are central to many physiological processes associated with health and disease, and highly diverse in nature.^11–13^ Several functional axes have been identified over the last years, e.g., the gut–brain axis, the gut–liver axis, the gut–lung axis, and the gut–immune axis.^14–17^ Moreover, direct and indirect effects of the human microbiome on bacterial infection are varied and complex^18,19^ (Figure 1). The human microbiota is increasingly recognized as a therapeutic target for infection prevention and treatment.

## Direct effects of microbial commensals: colonization resistance and pathobionts

Colonization resistance refers to the protection by the healthy microbiota against host colonization with pathogenic microorganisms. The search for the definition of a healthy microbiota has not been concluded, but a multitude of independent findings confirm that a high alpha diversity is associated with good health.^20,21^ The microbiota may however be a reservoir for potentially pathogenic commensals that can turn into causative agents of endogenic infections, so-called pathobionts. A loss in diversity or a disproportionate increase in one or more commensal species often indicates the presence of a disease state. Such shifts are commonly referred to as dysbiosis, even though an exact definition of this term remains to be established. The absence of dysbiosis plays a crucial role in the functionality of colonization resistance.

Individuals become colonized with multidrug-resistant (MDR) bacteria through contact with the healthcare system, the environment, animals, or the food chain. Initially, these bacteria may be present below the level of detection. However, exposure to antibiotics or other substances that exert selection pressure on the microbiota facilitates rapid expansion and domination of MDR bacteria. If these changes coincide with a breach in host barrier functions, e.g., in the context of chemotherapy or surgery, bacterial translocation, and infection with MDR bacteria become highly likely.

Different studies support this model of infection pathogenesis. Mice gavaged with vancomycin-resistant enterococci (VRE) prior to antibiotic exposure displayed functional colonization resistance with only minimal amounts of VRE detectable in their gut microbiota. If gavaged after antibiotic exposure, however, VRE was able to successfully colonize the gut even weeks after exposure. Apparently, some antibiotics are able to open a niche in the gut that favors the survival of VRE.^22^ With respect to Gram-negative MDR bacteria, an understanding of the niche required for the growth of MDR bacteria is less well established. Recent findings suggest that the synthesis of short-chain fatty acids (SCFA) by gut commensals may play an important role in this setting. While a balanced gut microbiota synthesizes enough SCFA to maintain an acidic pH in the gut, exposure to antibiotics induces a dysbiosis in the gut microbiota that leads to decreased SCFA production and an increase in pH. Under these circumstances, Gram-negative MDR bacteria are more likely to colonize and dominate the gut.^23^

## Indirect effects of microbial commensals: immune system regulation

The microbiome profoundly influences the host’s immune system. At birth, the innate and adaptive immune system is not yet fully developed. Interactions with microbes provide a central role in their development process by direct contact with commensal symbionts in addition to environmental antigens.^24^ During the first years of life of an infant, the early microbiota can shape the immune system and vice versa.^25^ The interactions between commensal bacteria and the human immune system are complex and their study is still in its infancy.

On the epithelial surface, the microbiota can regulate the integrity of the epithelial barrier, thereby preventing the penetration of pathogens into the host tissue and blood stream, and help respond to epithelial damage and pathogen breaches.^26,27^ This is mediated via pattern-recognition receptors (PRR) displayed in epithelial, endothelial, and immune cells, which detect microbe-associated molecular patterns (MAMPs) such as lipopolysaccharides and flagellin.^28,29^ Subsequent production of chemokines recruits immune cells, or activates the inflammasome.^27,30,31^ In turn, aspects of the inflammasome may influence the commensal microbiota.^32^ MAMPs are, however, not unique to pathogens, and it is unclear how commensals are distinguished from pathogens.^26^ Location may play an important role: commensals are mostly sequestrated on epithelial surfaces, while pathogens cross the epithelial barrier.^33,34^

Metabolites produced by bacterial commensals modulate innate and adaptive immune cells.^35^ A metabolite may have different effects depending on the receptor cell type, such as differentiation, activation, inhibition, migration, or production of antimicrobial peptides.^36–39^

Immune tolerance is the state of unresponsiveness of the immune system to agents that have the potential to induce an immune response.^40,41^ Intestinal regulatory T (Treg) cells play pivotal roles in the suppression of immune responses against harmless dietary antigens and commensal microorganisms.^42^ Differentiation, localization, and maintenance of intestinal Treg cells and tolerogenic dendritic cells are controlled by signals from the intestinal microbiota.^43^ Molecular mechanisms may involve PRR signaling or generation of microbial metabolites, but are still largely unidentified.

Finally, the commensal microbiota can contribute to dysregulation of the immune response. Bacterial dysbiosis has been associated with various diseases, among them asthma, allergies, obesity, chronic inflammatory disorders of the skin, colorectal cancer, and cardiovascular disease.^44–47^ In addition, dysbiosis of the commensal microbiota, such as caused by antibiotic treatment, has been associated with an increased risk of bloodstream infection and sepsis.^19,48^

## Alternative interventions against MDR pathogens

### Microbiota-based treatments

Based on our understanding of the key role of the microbiota in the prevention of colonization and infection with MDR bacteria, different microbiota-based treatments can be envisioned. Efforts to engineer or influence the commensal microbiota can be divided into two strategies: administration of live microorganisms, and supplementation with factors that influence the commensal microbiota.

#### Fecal microbiota transplantation

Fecal microbiota transplantation (FMT) is the transfer of stool from a healthy donor to the intestine of a patient. This can either be done endoscopically, by rectal enema, by oral ingestion of encapsulated preparations or by nasogastric or nasoduodenal tube. FMT is currently only recommended for the treatment of recurrent *Clostridioides difficile* infection (CDI), with an efficacy rate of up to 90%.^49,50^ How FMT decolonizes *C. difficile* has not been fully established, and microbial predictors of therapeutic outcome are not clear. Bile acid metabolism seems to play one of the central roles, however; the primary bile acid taurocholate, secreted by the liver, induces germination of *C. difficile* spores.^51^ Certain members of the commensal microbiota are able to metabolize primary bile acids and convert them into secondary bile acids, such as deoxycholate. While also able to induce germination, deoxycholate inhibits the vegetative growth of *C. difficile*.^51,52^ Other potential mechanisms for FMT efficacy may include microbiota modulation by direct interaction or competition (including quorum sensing modulation), and host immunity modulation.^53^

FMT is not currently approved for clinical use in the USA, but considered an investigational new drug.^54^ It is regulated individually in other countries.^55^ FMT is subject to safety concerns, namely transmission of infectious agents, including MDR pathogens,^56^ and unidentified risks associated with changes in the patient’s microbiota. Improved regulation, manufacturing standards, and stool banks are expected to mitigate the former.

In patients who receive FMT, a significant reduction in fecal bacterial antibiotic resistance genes was observed for Gram-positive pathogens,^57,58^ which suggests that FMT may harbor the potential to displace multiresistant bacteria from intestinal microbiota.^59^ Some clinical data are already available in this respect: in a recent study investigating the efficacy of a microbiota preparation as a treatment for recurrent CDI, successful VRE decolonization was recorded as a side effect.^60^ Similar results were documented in another small case study.^61^

With regard to decolonization of Gram-negative MDR bacteria, several case reports and uncontrolled studies, as well as one randomized trial assessing this question, provide mixed results to date.^62^ Application of FMT for MDR pathogen suppression in solid organ transplant recipients and patients with hematologic malignancies resulted in partial or full decolonization in multiple cases.^63^ Other recent anecdotal cases report eradication of MDR *Klebsiella pneumoniae* in a critically ill patient with endocarditis and sepsis originating from an infection of a pacemaker component,^64^ and MDR pathogen elimination in the case of cholangitis (inflammation of the bile duct system) with associated bacteremia.^65^

FMT is explored for treatment of other intestine-associated complications, such as inflammatory bowel disease, ulcerative colitis, and Crohn’s disease.^66^ Moreover, insights into the manifold effects of microbiota on human metabolism have spurred experimental FMT therapy for indications such as bipolar disorder (NCT03279224), Parkinson's disease (NCT03808389), cirrhosis (NCT02862249), and psoriatic arthritis (NCT03058900). Randomized controlled trials are currently ongoing, as well as experimental treatment of other diseases such as autoimmune diseases.^67^

#### Probiotics and live biotherapeutics

Probiotics are viable microorganisms which, when administered in sufficient quantities, have beneficial effects on the health of the host.^68^ If used as a drug with an associated health claim, they are referred to as live biotherapeutic products/agents.^69^ While probiotics are traditionally isolated from food, live biotherapeutics may be isolated from various niches. The latter may also include genetically modified organisms. Disease targets range from cancer, to autoimmune diseases (including asthma), to clearance of infectious agents. Mechanisms of action are specific to individual strains, and generally fall into one or multiple categories: microbiota modulation by direct interaction or competition, host metabolism modification,^11,12^ and host immunity modulation.

An example of a direct colonization resistance mechanism comes from *Staphylococcus lugdunensis*, a commensal isolated from the human nose.^70^ It produces lugdunin, a cyclic peptide antibiotic that inhibits the growth of various Gram-positive pathogens, including VRE and methicillin-resistant *Staphylococcus aureus*. Moreover, lugdunin amplifies the innate immune response by inducing expression of antimicrobial peptides and pro-inflammatory chemokines in human keratinocytes.^71^

Efforts are currently being made to identify defined bacterial consortia to suppress MDR strains.^18,72^ Importantly, administered strains must be free of antibiotic resistance genes.^73^ Results of randomized trials for MDR decolonization are mixed to date: two studies using *Lactobacillus rhamnosus* GG showed success in decolonizing patients with VRE.^74,75^ No effect on colonization was achieved against various Gram-negative MDR pathogens using a combination of *Lactobacillus bulgaricus* and *L. rhamnosus*.^76^

#### Prebiotics and postbiotics

Prebiotics are non-digestible food components that favorably influence human health by modulating the growth and/or activity of one or more species of commensals. Metabolites and cell components derived from probiotic strains, which influence the microbiota and host health, are referred to as postbiotics.^77^

Among the most commonly used prebiotics are oligosaccharides such as inulin, fructo-oligosaccharides and galacto-oligosaccharides: their fermentation by gut microbiota results in SCFA. Other classes of prebiotics are human milk oligosaccharides (HMO), conjugated linoleic acid and polyunsaturated fatty acids, polyphenols, and fermentable dietary fibers.^78,79^ The health benefits of prebiotics mostly depend on microbial utilization and the metabolites produced, rather than on parent compounds.^80^

Prebiotics may help prevent dysbiosis. In healthy volunteers, who were exposed to antibiotics, synthetically produced HMO positively influenced restoration of a balanced microbiota by selectively stimulating the growth of certain species, e.g., Actinobacteria and Bacteroidetes, while suppressing others, e.g., Firmicutes.^81^ HMO can also influence the innate immune response, and directly prevent adhesion of pathogens to the intestinal epithelium.^82,83^

Postbiotic metabolites comprise enzymes, proteins and peptides, lipids, polysaccharides, and organic acids. Postbiotic components include peptidoglycan, cell-surface proteins, exopolysaccharides, and teichoic acids. Postbiotics can act directly on the host (e.g. immunomodulation), the microbiota, or colonizing pathogens. In mice, extracellular vesicles from *Akkermansia muciniphila* and probiotic *Escherichia coli* increased the integrity of the epithelial gut barrier, contributing to one of the crucial factors that prevents systemic infection.^84,85^

### Phage-mediated therapies

Phages are viral bodies that infect bacteria via attachment to bacterial surface-proteins and introduce their own DNA into the bacterial genome. They exploit the bacterial transcription and translation machinery for the production of infectious particles. After assembly, phages exit via lysis,^86^ leading to cycles of reinfection and phage-mediated genome exchange between bacterial hosts. Microbiomes and phages are directly dependent on each other and in a state of continuous co-evolution.^87^ Coinfections of bacteria with multiple phages are the norm, resulting in a dynamic network of horizontal gene transfer that includes antibiotic resistance genes. While these gene transfers may result in the spread of resistance genes within a host, the mechanisms and the clinical relevance of these dynamics are topics of controversial discussions.^88–90^

The principle of bacterial genome-modification and lysis also holds therapeutic promise. D’Herelle et al. showed first-in-human application in *Vibrio cholerae* infection in the early twentieth century.^91,92^ Since then, the characterization of phages and phage–host interactions have been studied in depth to enable the translation of these findings into clinical practice.^93–95^ A key feature of phages is their high host specificity.^96^ Most known phages infect only a few strains of closely related bacterial populations, which leaves most of the commensal bacteria undisturbed. A variety of animal studies were able to show that phages can be used to eliminate MDR bacteria, including MDR *P. aeruginosa, A. baumannii*, and VRE.^97–99^

A number of smaller randomized controlled trials assessing the efficacy of phages in the treatment of bacterial infections have been published. While none of these studies reported any problems with respect to safety, response to treatment was inconsistent between studies.^100–105^ Next to further improvement of efficacy, a regulatory framework for phage therapies needs to be put in place.

### Vaccines and antibodies

Vaccines and antibodies are designed to prevent infection or to decrease disease severity. Effective vaccines deliver antigens to elicit a prophylactic immune response to generate disease-specific antibodies with their corresponding memory cells, and provide long-term protection against invading pathogens.^106,107^ Externally administered antibodies are agents of passive immunization, act faster than vaccines (hours or days), and bestow short-term protection.^108^

Neither vaccines nor antibody preparations specifically act against MDR bacterial strains to date, but they can reduce the spread of targeted pathogenic strains.^109^ This may decrease the number of antibiotics used, which reduces the selection pressure on antibiotic resistance, aligns with antimicrobial stewardship measures, and forgoes antibiotic de-colonization of commensal microbiota. By avoiding antibiotic treatment, vaccines can potentially reduce bystander selection of resistance elements in the commensal microbiota: the proliferation of bacteria carrying resistance genes upon targeted antibiotic removal of a neighboring pathogenic species.^110,111^

Resistance development to vaccines is relatively infrequent compared to the emergence of antibiotic resistance,^112^ but serotype replacement followed by the spread of new MDR serotypes have been observed,^113^ as well as an increase in invasive, non-vaccine serotype strains of targeted pathogens (e.g. *Haemophilus influenzae, Streptococcus pneumoniae*).^114–116^ Pathogenic strains are often heterogeneous and diverse, which increases the complexity of vaccine development.^117^ Moreover, antibiotic resistance mechanisms are frequently encoded in mobile genetic elements and horizontally transferred.^118,119^ Vaccines directed at the effectors of drug resistance, such as penicillin-binding proteins and β-lactamases, are being studied in animal models.^120,121^ Vaccines against gut-associated bacterial pathogens are currently available for *Vibrio cholerae* (Vaxchora), *Salmonella typhi* (Vivotif Berna), as well as *Bacillus anthracis* (BioThrax).

The commensal microbiota was shown to influence vaccine efficacy. Flagellin derived from commensal microbiota, for instance, may play an adjuvant role and enhance immune response in response to vaccination.^122,123^ Components of lipoteichoic acid and peptidoglycan appear to have similar effects.^124,125^

### Mitigating antibiotic collateral damage

Approximately, a quarter of all inpatients treated at hospitals receive antibiotics. One-third to one-half of antibiotic prescriptions in inpatient settings are insufficient with regard to the indication and/or duration of therapy.^126,127^ This fosters selection for antibiotic resistance and the spread of nosocomial MDR strains. The use of broad-spectrum antibiotics can devastate the beneficial commensal microbiota, thus rendering a patient temporarily more susceptible to opportunistic infections, induce dysbiosis, and even cause long-lasting complications such as asthma and inflammatory bowel disease.^128–130^ Potential solutions to address this issue are the development of treatment options with increased specificity or confined activity. Examples are narrow-spectrum antibiotics,^131^ selective pathogen-targeting phages^96^ or antibody-antibiotic conjugates,^132^ localized antibiotic delivery using nanoparticle formulations,^133^ and strategies to protect the gut microbiota with orally administered beta-lactamases^134^ or slow-release formulations of activated charcoal that absorb antibiotics as soon as they reach the large intestine.^134^

### Antimicrobial stewardship

Antimicrobial stewardship (AMS) programs constitute a vital strategy to address antimicrobial resistance. They are designed to improve the quality of antimicrobial prescriptions in terms of substance selection, dosage, route of administration, and duration of therapy. AMS has been shown to reduce adverse events such as sepsis, results in lower mortality rates and improves patient outcomes, and decreases the rate of colonization and infection with MDR bacteria.^135–138^ The above outlined potential alternative therapies are not only fully compatible with AMS strategies, but would facilitate its implementation and potentially enhance its effectiveness.

## Conclusion and outlook

As the antibiotic resistance crisis is unfolding, it has become clear that we cannot rely on classic antibiotics alone to suppress the rise of MDR bacterial pathogens. While antibiotic stewardship and the development of single-component molecule antibiotics are of utmost importance, we will increasingly have to rely on alternative strategies to support or even replace conventional antibiotic treatment. Vaccines and phages have already shown great promise in past applications and proof-of-concept studies. Our greatest ally may yet become the human commensal microbiota. While it is only recently that we began to systematically uncover the myriad ways in which these microorganisms contribute to health and disease, it is already evident that they offer many intervention points to combat infectious agents directly or in tandem with the human host. We have a lot of work ahead of us to attain safe and efficacious treatments, and it is most certainly exciting and promising.
